# Abdominal compartment syndrome as a complication of endoscopic carbon dioxide insufflation in a patient with malignant bowel obstruction: a case report

**DOI:** 10.1186/s40792-023-01783-9

**Published:** 2023-11-21

**Authors:** Taro Tanabe, Genki Tsukuda, Takahiro Hobo, Noboru Yokoyama, Haruhiro Inoue

**Affiliations:** Digestive Diseases Center, Showa University Koto Toyosu Hospital, 5-1-38 Toyosu, Koto-ku, Tokyo, 135-8577 Japan

**Keywords:** Abdominal compartment syndrome, Self-expandable metal stent, Colonic obstruction, Pneumoperitoneum, Case report

## Abstract

**Background:**

A self-expandable metal stent is often placed as a bridge to elective surgical treatment of left-sided malignant obstruction of the colon because it allows for primary anastomosis without the need for a temporary stoma, which has a positive impact on the patient’s quality of life. However, although a relatively safe procedure, colonic stenting can have complications that require emergency surgery. This case report describes a rare case of abdominal compartment syndrome that occurred as a complication of endoscopic insufflation during colonic stenting.

**Case presentation:**

The patient was a 72-year-old woman who presented complaining of several days of constipation and loss of appetite. Computed tomography of the abdomen revealed obstruction of the sigmoid colon by a tumor. There were no symptoms or computed tomography findings to suggest perforation. Therefore, an attempt was made to insert a self-expandable metal stent. Acute respiratory disturbance and a change in consciousness occurred during the stenting procedure, with marked abdominal distention. Abdominal compartment syndrome was diagnosed and treated by decompressive laparotomy.

**Conclusions:**

To the best of our knowledge, this is the first reported case of abdominal compartment syndrome as a complication of endoscopic insufflation during colonic stenting. The possibility of abdominal compartment syndrome should be considered if acute respiratory disturbance or altered consciousness occurs during endoscopic procedure in a patient with malignant bowel obstruction.

## Background

Self-expandable metal stent (SEMS) implantation is being used increasingly in the management of malignant colorectal obstruction, not only for palliative purposes, but also for preoperative treatment in patients who are candidates for surgery [1]. The clinical guidelines for obstructing colonic and extracolonic cancer published by the European Society of Gastrointestinal Endoscopy in 2014 did not recommend insertion of a SEMS as a bridge to surgery in a patient with potentially curable left-sided obstructive colon cancer [2]; however, the 2020 update to the guidelines recommends stent insertion as an alternative to emergency resection [3] because of its increasing success rate and a decrease in the rate of early complications [4]. Although stenting of the colon is considered a relatively safe procedure with a mortality rate of approximately 1% [5], it can have important complications that require emergency surgery [4–6].

Abdominal compartment syndrome (ACS) is defined as sustained intra-abdominal pressure above 20 mmHg with new onset of organ dysfunction [7]. The major risk factors for ACS are large-volume fluid resuscitation for sepsis, shock, and other inflammatory conditions, such as pancreatitis [8].

We have encountered a case of ACS without perforation caused by tension pneumoperitoneum that occurred because of carbon dioxide insufflation during stenting for malignant colonic obstruction.

## Case presentation

**Fig. 1:**
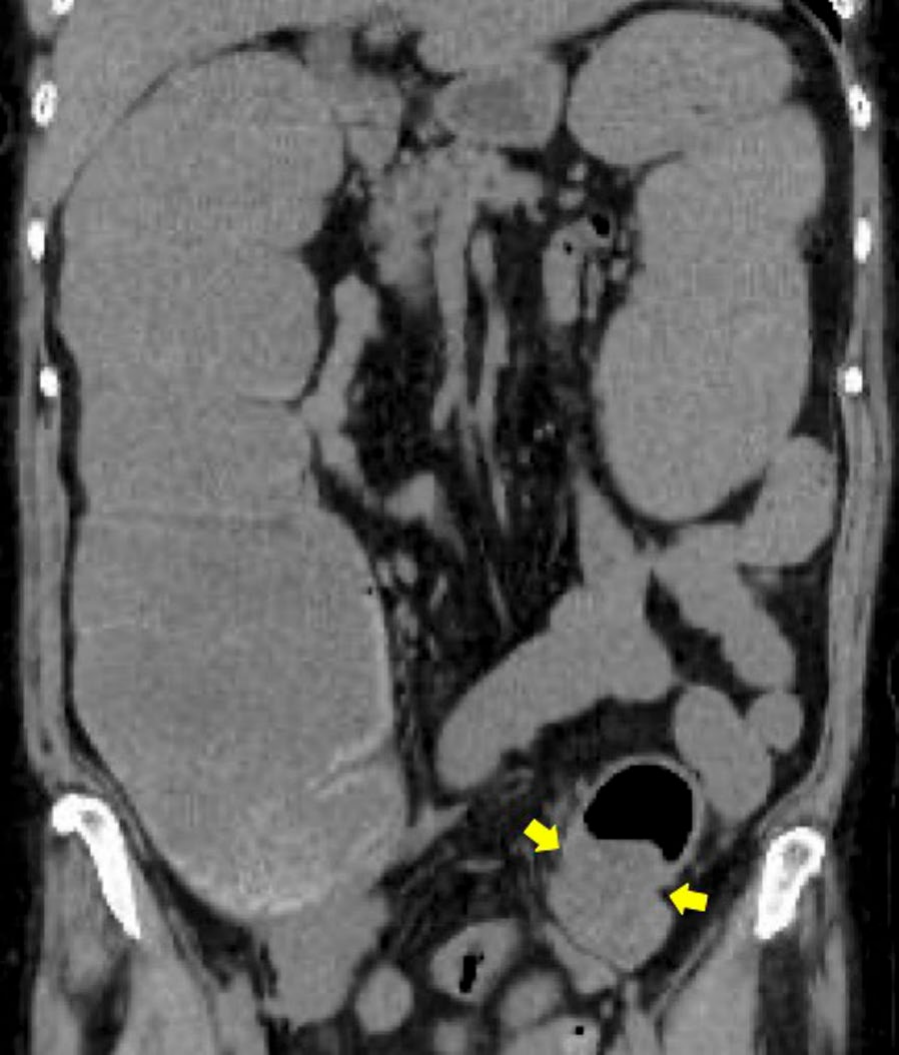
Computed tomography scan obtained before colonic stenting. The scan shows wall thickening in the sigmoid colon (yellow arrows) and dilatation of the colon and small intestine on the oral side of it

A 72-year-old woman (height, 148cm; weight, 50kg; BMI, 22.8) presented to our hospital complaining of constipation and loss of appetite in the previous few days. She did not appear to be unwell with a blood pressure of 128/75 mmHg, a heart rate 93 beats/min, a body temperature of 36.6 °C, and a respiratory rate of 22 breaths/min with 99% oxygen saturation in room air. Her Glasgow Coma Scale (GCS) was E4V5M6. Physical examination revealed a distended but non-tender abdomen. Laboratory investigations did not reveal any obvious abnormalities. A computed tomography (CT) scan confirmed marked colonic dilatation caused by cancer in the sigmoid colon (Fig. 1). There were no metastatic lesions. The diagnosis was obstructive sigmoid colon cancer without impending sepsis. The decision was made to insert a SEMS.

**Fig. 2:**
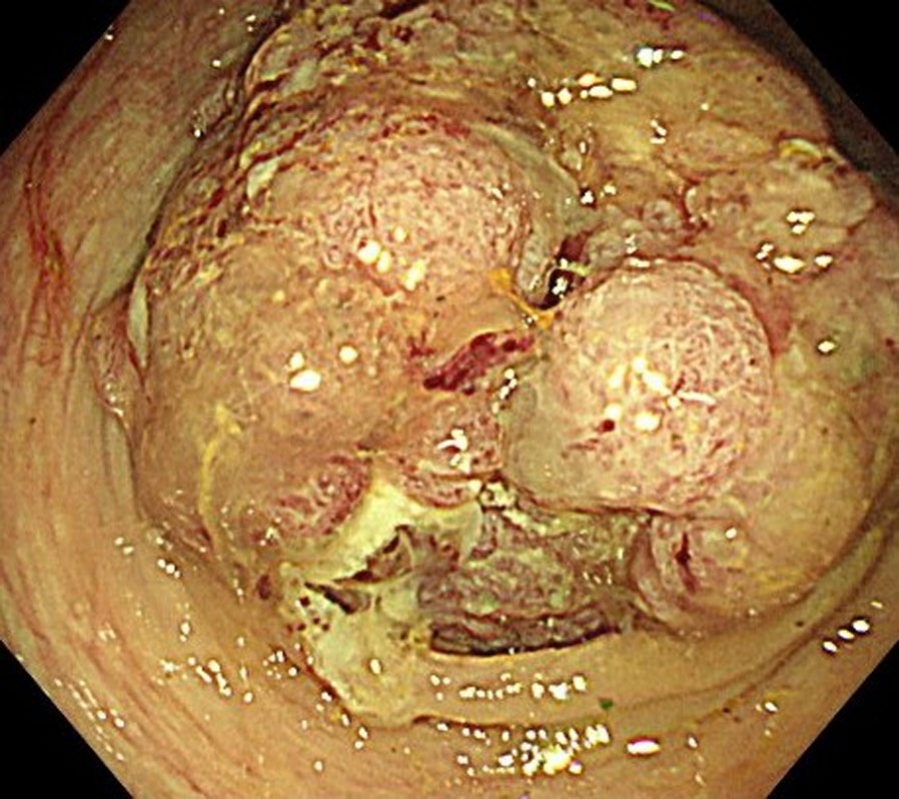
Colonoscopic image obtained at the time of colonic stenting. The image shows a full circumferential tumor that was almost completely occluding the sigmoid colon, making it difficult to pass a guidewire

**Fig. 3:**
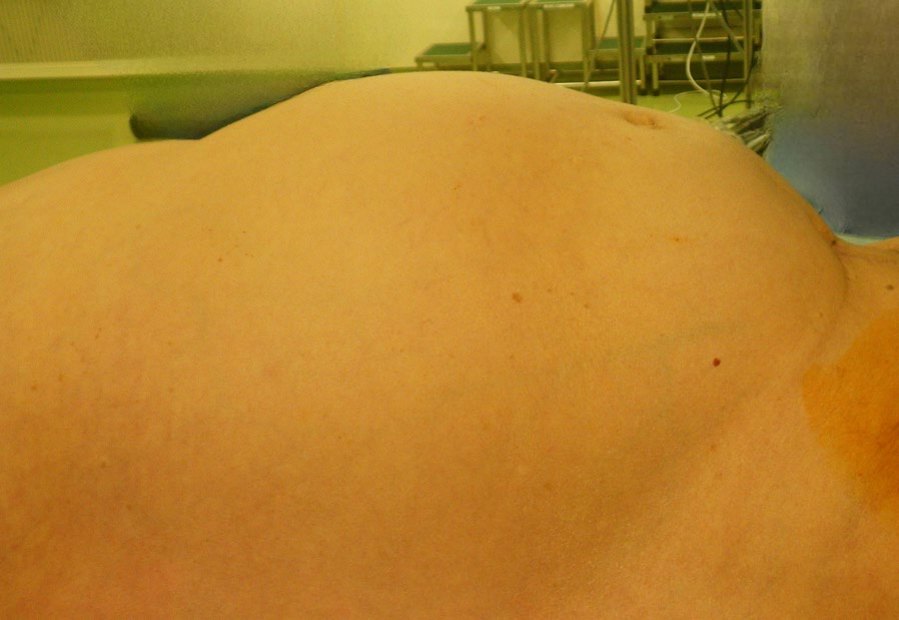
Physical examination on completion of colonic stenting revealed a significantly distended abdomen

**Fig. 4:**
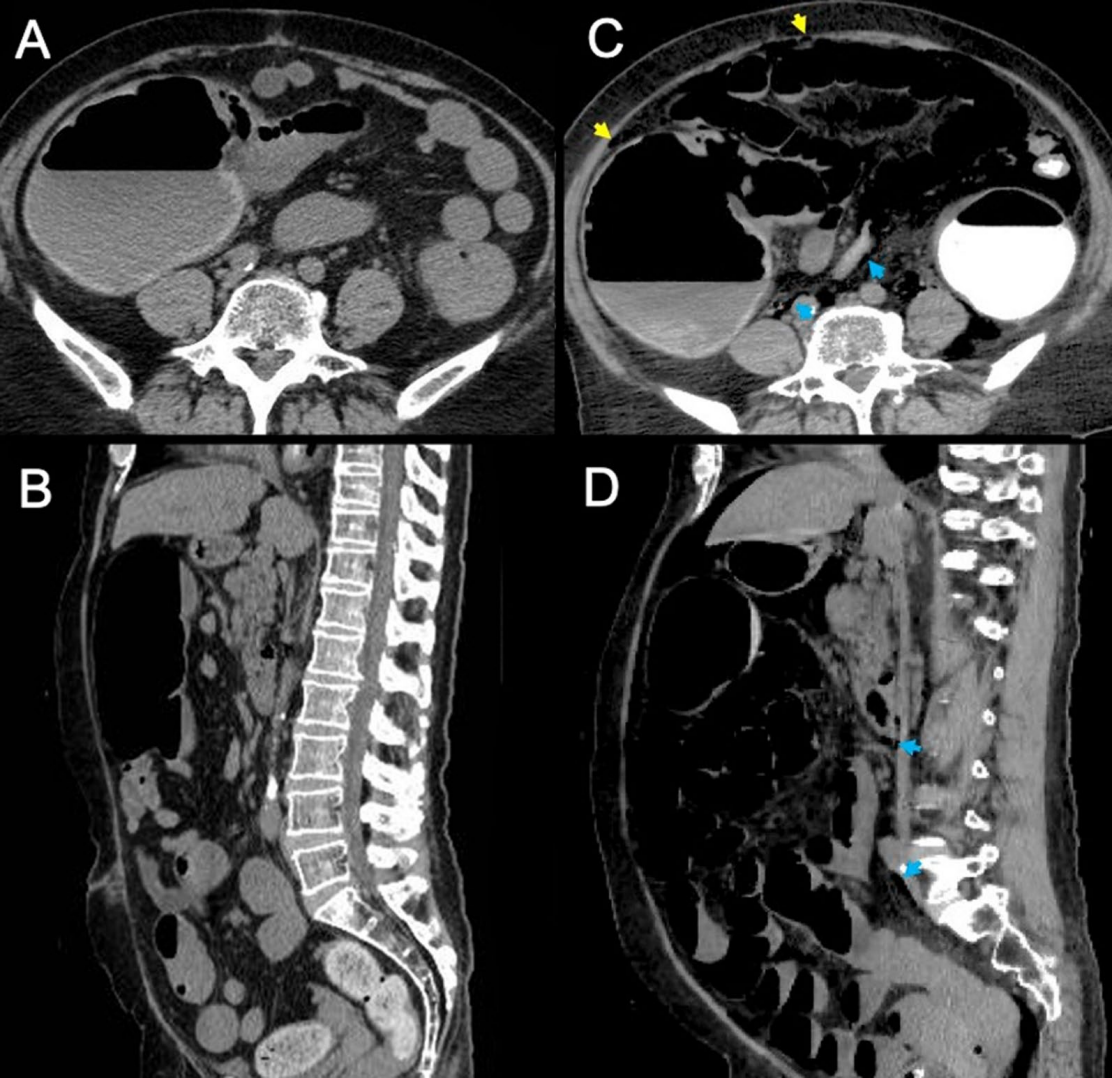
Computed tomography scans obtained after and before colonic stenting. **a** An axial view before colonic stenting. **b** A sagittal view before colonic stenting. **c** An axial view after colonic stenting shows the presence of air in the abdominal cavity (yellow arrows) and retroperitoneal cavity (blue arrows), mainly in the mesentery of the sigmoid colon, and dilatation of the colon without spillage of contrast. **d** A sagittal view shows marked distention of the abdomen caused by accumulation of gas in the small intestine and colon

Endoscopy showed a full circumferential tumor that was almost completely occluding the sigmoid colon and making it difficult to pass a guidewire (Fig. 2). Forty minutes after the start of endoscopy, the patient became cyanotic, unresponsive, and developed a severely distended abdomen (Fig. 3) with a blood pressure of 69/42 mmHg, a heart rate 50 beats/min, and a respiratory rate of 33 breaths/min with 59% oxygen saturation. Her level of consciousness and oxygenation did not improve after withdrawal of sedation and immediate termination of the insertion procedure. The patient was emergently intubated, but ventilation was difficult, her tidal volume was under 200 mL with pressure support of 15 cm H2O. Urgent CT, compared to pre-stent insertion (Fig. 4A and B), demonstrated marked bowel dilatation and pneumoperitoneum without spillage of contrast (Fig. 4C and D).

Based on the marked abdominal dilatation, difficulty ventilation, and CT findings, a diagnosis of ACS was made clinically, and emergency decompressive laparotomy was performed.

**Fig. 5:**
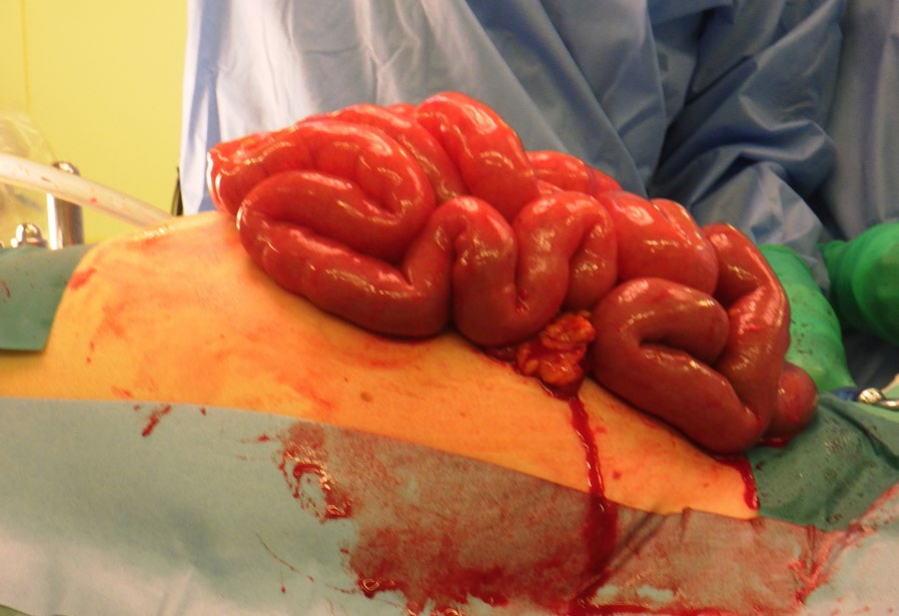
Clinical photograph obtained at the time of emergency laparotomy. The image shows overflow from a markedly dilated intestinal tract and the wound immediately after the abdomen is opened. There is no obvious intestinal perforation or intra-abdominal contamination

Almost immediately after the laparotomy, the markedly dilated intestinal tract was ejected from the body cavity, and respiratory status improved (Fig. 5). There was no obvious intestinal perforation or contamination of the abdominal cavity. Based on the intraoperative findings, the dilated intestinal tract due to insufflation during stenting procedure was the cause of the ACS, and the colon was decompressed by resecting the appendix and aspirating the intestinal contents at the same site, followed by a Hartmann’s procedure.

The patient’s condition Improved promptly after surgery. She was extubated on the day after surgery, discharged from the intensive care unit on the following day and discharged from hospital about two months after surgery. There was no functional impairment discovered at the outpatient clinic after 6 months of discharge.

## Discussion

This report has two important clinical teaching points: (1) ACS can occur as a complication of endoscopic carbon dioxide insufflation and (2) ACS should be suspected if a sudden change in level of consciousness or respiratory disturbance occurs during endoscopic procedure in a patient with malignant bowel obstruction.

Colonic stenting as a bridge to surgery is recommended as one of the treatment options for malignant colonic obstruction in the European guidelines [3]. However, while colonic stenting is considered a relatively safe procedure, the stent-related mortality rate is approximately 1% [9], and complications that require emergency surgery, such as colonic perforation, occur in 3.7% to 4.8% of cases [6, 9–11]. Therefore, candidates for colonic stenting should be selected carefully, given that they are often already in poor condition.

This is the first report of ACS as a complication of endoscopic insufflation during colonic stenting. In this case, the sigmoid colon cancer acted as a one-way valve, trapping the air delivered by the colonoscope and causing remarkable intestinal dilation and pneumoperitoneum without perforation, resulting in ACS. Pneumoperitoneum has been reported to occur at rates of 0.3%–1% in diagnostic colonoscopy and 3% in therapeutic colonoscopy [12]. Most cases of pneumoperitoneum without perforation do not require surgical intervention; however, there has been a report of a case of ACS caused by pneumoperitoneum without perforation [13]. In patients with malignant colonic obstruction, intra-abdominal pressure can be high even before colonoscopy is started; therefore, careful attention to air delivery is needed during colonic stenting.

Perforation at the time of stent insertion is a major complication requiring surgery and often manifests as abdominal pain or subcutaneous emphysema one hour to one day later [6, 14]. However, in a case of ACS, acute respiratory failure and a change in consciousness occurs during the procedure. Therefore, ACS should be suspected when these changes occur during stent insertion. Marked abdominal dilatation on physical examination is important for diagnosis, and pneumoperitoneum without contrast leak on CT may be a useful finding.

ACS is usually caused by rapid addition of volume [15], in which case primary abdominal closure after decompressive laparotomy is often difficult [7, 16, 17]. However, when ACS is caused by endoscopic manipulation, primary abdominal closure can be achieved because gas is the main cause of the increase in intra-abdominal pressure.

## Conclusions

We have reported the first case of ACS as a complication of endoscopic carbon dioxide insufflation during colonic stenting. Acute respiratory failure or change in consciousness during this procedure should raise suspicion for ACS.

## Data Availability

All data on which the conclusions of this case report are based are included in the present publication.

## Abbreviations

ACS: Abdominal compartment syndrome
CT: Computed tomography
SEMS: Self-expandable metal stent
